# Methyl 2-[(*E*)-(4-nitro­phen­yl)hydrazono]-3-oxobutyrate

**DOI:** 10.1107/S160053680802312X

**Published:** 2008-07-26

**Authors:** Yong-Hong Liu, Gui-You Sun, Jian-Feng Liu, Jun Ye, Xiao-Lan Liu

**Affiliations:** a College of Chemistry and Chemical Engineering, Yangzhou University, Yangzhou 225002, People’s Republic of China; b Technology Center, Jiuquan Iron and Steel (Group) Co. Ltd., Jiayuguan 735100, People’s Republic of China

## Abstract

The mol­ecule of the title compound, C_11_H_11_N_3_O_5_, exists as the *E* isomer as it is stabilized by an intra­molecular hydrogen bond. Except for the methyl H atoms, all atoms lie in special positions on a mirror plane and form a large conjugated system; the methyl H atoms are disordered about the mirror plane. In the crystalline state, bifurcated intra- and inter­molecular N—H⋯O hydrogen bonds and four inter­molecular C—H⋯O hydrogen bonds link the mol­ecules into large perfectly planar sheets. Along the *c* axis, the N—N bond center approaches the phenyl-ring centroids of its neighbouring mol­ecules above and below to give π–π overlap (at a distance of *ca* 3.57 Å), thus fusing the mol­ecules into a three-dimensional framework.

## Related literature

For related literature, see: Bernstein *et al.* (1995 ▶); Lewis *et al.* (1999 ▶); Liu *et al.* (2007 ▶, 2008 ▶); Mague *et al.* (1997 ▶); Mahy *et al.* (1993 ▶); Serbutoviez *et al.* (1995 ▶); Thami *et al.* (1992 ▶); Wang *et al.* (2005 ▶).
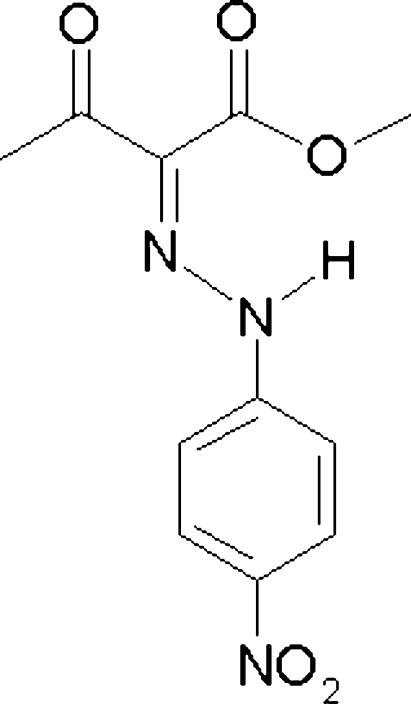


## Experimental

### 

#### Crystal data

C_11_H_11_N_3_O_5_
*M* *_r_* = 265.2
Orthorhombic, 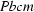

*a* = 12.880 (3) Å
*b* = 14.299 (3) Å
*c* = 6.6328 (14) Å
*V* = 1221.6 (5) Å^3^
*Z* = 4
Mo *K*α radiation
μ = 0.12 mm^−1^
*T* = 296 (2) K
0.30 × 0.30 × 0.20 mm

#### Data collection

Bruker SMART 1000 CCD diffractometer
Absorption correction: multi-scan (*SADABS*; Bruker, 2002 ▶) *T*_min_ = 0.966, *T*_max_ = 0.977
10245 measured reflections
1546 independent reflections
968 reflections with *I* > 2σ(*I*)
*R*_int_ = 0.041

#### Refinement

*R*[*F* ^2^ > 2σ(*F* ^2^)] = 0.042
*wR*(*F* ^2^) = 0.121
*S* = 1.03
1546 reflections
118 parameters
H-atom parameters constrained
Δρ_max_ = 0.20 e Å^−3^
Δρ_min_ = −0.13 e Å^−3^

### 

Data collection: *SMART* (Bruker, 2002 ▶); cell refinement: *SAINT* (Bruker, 2002 ▶); data reduction: *SAINT*; program(s) used to solve structure: *SHELXS97* (Sheldrick, 2008 ▶); program(s) used to refine structure: *SHELXL97* (Sheldrick, 2008 ▶); molecular graphics: *PLATON* (Spek, 2003 ▶); software used to prepare material for publication: *SHELXTL* (Sheldrick, 2008 ▶).

## Supplementary Material

Crystal structure: contains datablocks I, global. DOI: 10.1107/S160053680802312X/cs2083sup1.cif

Structure factors: contains datablocks I. DOI: 10.1107/S160053680802312X/cs2083Isup2.hkl

Additional supplementary materials:  crystallographic information; 3D view; checkCIF report

## Figures and Tables

**Table 1:** Hydrogen-bond geometry (Å, °)

*D*—H⋯*A*	*D*—H	H⋯*A*	*D*⋯*A*	*D*—H⋯*A*
N1—H1⋯O4	0.86	1.98	2.618 (3)	130
C2—H2⋯O3^i^	0.93	2.55	3.279 (3)	135
C11—H11*B*⋯O1^ii^	0.96	2.57	3.128 (3)	117
N1—H1⋯O2^iii^	0.86	2.64	3.439 (3)	154
C5—H5⋯O2^iii^	0.93	2.62	3.467 (4)	153
C4^iii^—H4^iii^⋯O4	0.93	2.61	3.518 (3)	167

Symmetry codes: (i) 
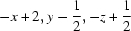
; (ii) 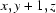; (iii) 
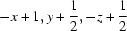
.

**Fig. 1.**
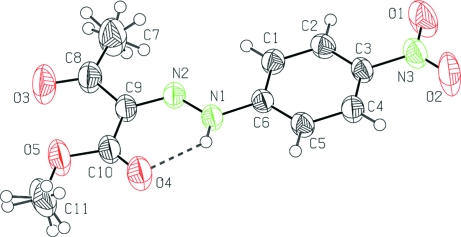
The molecular structure of the title compound, showing 30% probability displacement ellipsoids. Disorder of the two methyl groups are indicated and the N–H···O intra-molecular hydrogen bond shown as dashed lines.

**Fig. 2.**
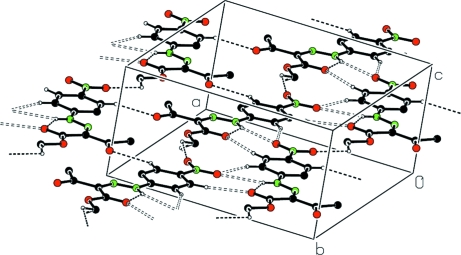
Part of the crystal structure of the title compound, showing the formation of a hydrogen bonded plane parallel to [001], which is built by one N—H···O and four C—H···O inter-molecular hydrogen bonds (dashed lines). For the sake of clarity, H atoms not involved in hydrogen bonding have been omitted.

**Fig. 3.**
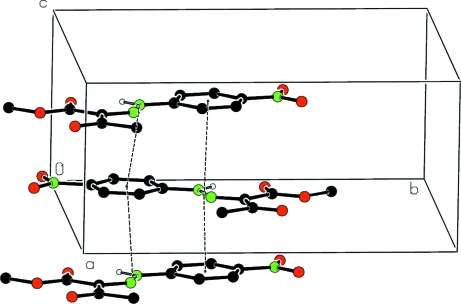
Excerpt of the crystal structure of the title compound, showing that along the c axis the N1—N2 bond center of one molecule combines its up and down phenyl rings in the other two molecules into a three dimensional framework. H atoms not involved in hydrogen bonding have been omitted.

**Fig. 4.**
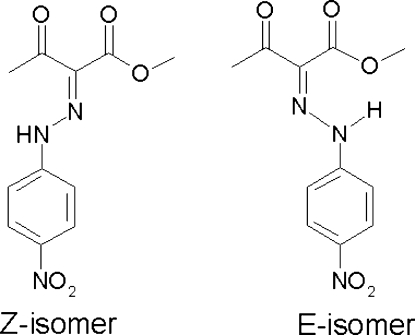
The E and Z isomers of the title compound.

## supplementary crystallographic information

### Comment

Phenylhydrazone and its derivatives show remarkable stability and high tendency to form non-centrosymmetric crystal packing (Lewis *et al.*, 1999; Mague *et al.*, 1997) and exceptional electronic, bioactive and chemical properties useful for analytic purposes (Mahy *et al.*, 1993), for biological chemistry (Thami *et al.*, 1992) and also for optical materials (Serbutoviez *et al.*, 1995). As a part of our ongoing research (Liu *et al.*, 2007; Liu *et al.*, 2008), the crystal structure of the title compound was solved.

The molecule of the title compound exists in the (*E*)-isomer configuration, not as the generally more stable (*Z*)-isomer (Schemes 1 and 2). The (*E*)-isomer exists here because of the N—H···O intra-molecular hydrogen bond stabilizes it by forming a pseudo-ring *S*(6) (Bernstein *et al.*, 1995) motif (Fig. 1, Table 1 and 2). The N1—C6 bond distance at 1.397 (3) Å is longer than the expected C═N double bond (1.32 Å) but is shorter than a C—N single bond (1.47 Å) because of the classic *sp*^2^-hybrid nitrogen atom, as also found in our earlier work (Liu *et al.*, 2007, 2008). All these effects may help all non-hydrogen atoms to form a perfect plane which coincides with the mirror plane of the space group, less for the hydrogen atoms of the two methyl groups whose six H atoms are disordered over two orientations.

In the crystal packing the molecules are linked into larger perfectly planar sheets *via* by four C—H···O inter-molecular hydrogen bonds and one N—H···O intra-molecular hydrogen bond running parallel to the [001] plane (Fig. 2, Table 2). H1 atom of the N1 atom is a part of a bifurcated system and makes both intra- and intermolecular H-bridges, with angles around the H1 adding up to 360°. Finally, along the *c* axis the N1—N2 bond centers of molecules combine its up and down neighbours' phenyl rings into three dimensional framework (Fig. 2). Consecutive bond centers···phenyl ring centers are at a distance of ca. 3.57 Å and an incline at an angle of ca. 137° (Fig. 3).

### Experimental

The title compound was synthesized according to literature procedure (Wang *et al.* 2005; Liu *et al.* 2008). Crystals suitable for X-ray diffraction were obtained by slow evaporation of a solution of the solid in dichloromethane at room temperature over a period of 6 d.

### Refinement

After their location in a difference map, all H atoms were fixed geometrically at ideal positions and allowed to ride on the parent C atoms, with C—H distances of 0.93 (aromatic) or 0.97 Å (methyl), and with *U_iso_*(H) values of *1.2U_eq_* (C, N).

### Crystal data

**Table:**

C_11_H_11_N_3_O_5_	*D*_x_ = 1.442 Mg m^−^^3^
*M**_r_* = 265.2	Melting point: 400 K
Orthorhombic, *P**b**c**m*	Mo *K*α radiation λ = 0.71073 Å
Hall symbol: -P 2c 2b	Cell parameters from 1996 reflections
*a* = 12.880 (3) Å	θ = 2.8–25.4º
*b* = 14.299 (3) Å	µ = 0.12 mm^−^^1^
*c* = 6.6328 (14) Å	*T* = 296 (2) K
*V* = 1221.6 (5) Å^3^	Block, yellow
*Z* = 4	0.30 × 0.30 × 0.20 mm
*F*_000_ = 552	

### Data collection

**Table:**

Bruker SMART 1000 CCD diffractometer	1546 independent reflections
Radiation source: fine-focus sealed tube	968 reflections with *I* > 2σ(*I*)
Monochromator: graphite	*R*_int_ = 0.041
*T* = 296(2) K	θ_max_ = 27.6º
Thin–slice ω scans	θ_min_ = 1.6º
Absorption correction: multi-scan(SADABS; Bruker, 2002)	*h* = −16→16
*T*_min_ = 0.966, *T*_max_ = 0.977	*k* = −17→18
10245 measured reflections	*l* = −8→8

### Refinement

**Table:**

Refinement on *F*^2^	Hydrogen site location: inferred from neighbouring sites
Least-squares matrix: full	H-atom parameters constrained
*R*[*F*^2^ > 2σ(*F*^2^)] = 0.042	*w* = 1/[σ^2^(*F*_o_^2^) + (0.0497*P*)^2^ + 0.2977*P*] where *P* = (*F*_o_^2^ + 2*F*_c_^2^)/3
*wR*(*F*^2^) = 0.121	(Δ/σ)_max_ < 0.001
*S* = 1.03	Δρ_max_ = 0.20 e Å^−^^3^
1546 reflections	Δρ_min_ = −0.13 e Å^−^^3^
118 parameters	Extinction correction: SHELXL97 (Sheldrick, 2008), Fc^*^=kFc[1+0.001xFc^2^λ^3^/sin(2θ)]^-1/4^
Primary atom site location: structure-invariant direct methods	Extinction coefficient: 0.0036 (9)
Secondary atom site location: difference Fourier map	

### Fractional atomic coordinates and isotropic or equivalent isotropic displacement parameters (Å^2^)

**Table:**

	*x*	*y*	*z*	*U*_iso_*/*U*_eq_	Occ. (<1)
O4	0.68298 (15)	0.51128 (12)	0.2500	0.0726 (6)	
O3	1.00596 (16)	0.52888 (14)	0.2500	0.0896 (8)	
O1	0.68240 (17)	−0.10594 (13)	0.2500	0.0887 (8)	
O2	0.52310 (17)	−0.06963 (13)	0.2500	0.1081 (10)	
N3	0.61352 (18)	−0.04820 (14)	0.2500	0.0582 (6)	
N1	0.72021 (14)	0.33126 (12)	0.2500	0.0422 (5)	
H1	0.6721	0.3730	0.2500	0.051*	
N2	0.81817 (14)	0.35582 (13)	0.2500	0.0435 (5)	
C10	0.7757 (2)	0.52229 (16)	0.2500	0.0489 (6)	
C6	0.69506 (16)	0.23623 (15)	0.2500	0.0379 (5)	
C8	0.9648 (2)	0.45339 (18)	0.2500	0.0597 (7)	
C5	0.59053 (17)	0.21060 (14)	0.2500	0.0436 (6)	
H5	0.5392	0.2563	0.2500	0.052*	
C2	0.74469 (18)	0.07505 (15)	0.2500	0.0447 (6)	
H2	0.7957	0.0290	0.2500	0.054*	
C4	0.56352 (18)	0.11723 (15)	0.2500	0.0475 (6)	
H4	0.4941	0.0993	0.2500	0.057*	
O5	0.81917 (15)	0.60575 (12)	0.2500	0.0768 (7)	
C3	0.64146 (18)	0.05094 (15)	0.2500	0.0423 (5)	
C1	0.77167 (17)	0.16807 (15)	0.2500	0.0431 (6)	
H1A	0.8413	0.1853	0.2500	0.052*	
C9	0.84922 (18)	0.44293 (16)	0.2500	0.0453 (6)	
C7	1.0288 (2)	0.3663 (2)	0.2500	0.0906 (12)	
H7A	1.0318	0.3412	0.1158	0.136*	0.50
H7B	0.9980	0.3211	0.3387	0.136*	0.50
H7C	1.0978	0.3806	0.2954	0.136*	0.50
C11	0.7477 (3)	0.68338 (19)	0.2500	0.0908 (11)	
H11A	0.7153	0.6882	0.1201	0.136*	0.50
H11B	0.7847	0.7401	0.2788	0.136*	0.50
H11C	0.6955	0.6735	0.3511	0.136*	0.50

### Atomic displacement parameters (Å^2^)

**Table:**

	*U*^11^	*U*^22^	*U*^33^	*U*^12^	*U*^13^	*U*^23^
O4	0.0474 (12)	0.0424 (10)	0.1281 (19)	−0.0013 (8)	0.000	0.000
O3	0.0517 (12)	0.0553 (13)	0.162 (2)	−0.0184 (10)	0.000	0.000
O1	0.0783 (14)	0.0355 (10)	0.152 (2)	0.0124 (10)	0.000	0.000
O2	0.0591 (13)	0.0402 (11)	0.225 (3)	−0.0140 (10)	0.000	0.000
N3	0.0587 (14)	0.0335 (11)	0.0824 (16)	−0.0003 (11)	0.000	0.000
N1	0.0398 (10)	0.0318 (10)	0.0551 (12)	−0.0030 (8)	0.000	0.000
N2	0.0430 (11)	0.0392 (10)	0.0482 (12)	−0.0067 (9)	0.000	0.000
C10	0.0524 (16)	0.0355 (13)	0.0588 (16)	−0.0074 (11)	0.000	0.000
C6	0.0426 (12)	0.0333 (11)	0.0377 (12)	−0.0018 (10)	0.000	0.000
C8	0.0493 (15)	0.0478 (15)	0.0821 (19)	−0.0102 (13)	0.000	0.000
C5	0.0417 (12)	0.0317 (12)	0.0575 (14)	0.0039 (9)	0.000	0.000
C2	0.0434 (13)	0.0358 (12)	0.0547 (14)	0.0065 (10)	0.000	0.000
C4	0.0392 (12)	0.0369 (12)	0.0664 (16)	−0.0023 (10)	0.000	0.000
O5	0.0622 (12)	0.0348 (10)	0.1333 (19)	−0.0092 (9)	0.000	0.000
C3	0.0456 (13)	0.0283 (11)	0.0530 (14)	0.0000 (10)	0.000	0.000
C1	0.0381 (12)	0.0404 (13)	0.0507 (14)	−0.0020 (10)	0.000	0.000
C9	0.0460 (13)	0.0360 (12)	0.0537 (14)	−0.0081 (10)	0.000	0.000
C7	0.0511 (17)	0.0568 (17)	0.164 (4)	−0.0011 (14)	0.000	0.000
C11	0.088 (2)	0.0336 (14)	0.150 (3)	−0.0002 (16)	0.000	0.000

### Geometric parameters (Å, °)

**Table:**

O4—C10	1.204 (3)	C5—C4	1.380 (3)
O3—C8	1.203 (3)	C5—H5	0.9300
O1—N3	1.212 (3)	C2—C3	1.374 (3)
O2—N3	1.204 (3)	C2—C1	1.375 (3)
N3—C3	1.463 (3)	C2—H2	0.9300
N1—N2	1.310 (2)	C4—C3	1.381 (3)
N1—C6	1.397 (3)	C4—H4	0.9300
N1—H1	0.8600	O5—C11	1.442 (3)
N2—C9	1.308 (3)	C1—H1A	0.9300
C10—O5	1.318 (3)	C7—H7A	0.9600
C10—C9	1.478 (3)	C7—H7B	0.9600
C6—C1	1.387 (3)	C7—H7C	0.9600
C6—C5	1.395 (3)	C11—H11A	0.9600
C8—C7	1.494 (4)	C11—H11B	0.9600
C8—C9	1.496 (3)	C11—H11C	0.9600
			
O2—N3—O1	122.3 (2)	C3—C4—H4	120.6
O2—N3—C3	119.0 (2)	C10—O5—C11	115.2 (2)
O1—N3—C3	118.7 (2)	C2—C3—C4	122.1 (2)
N2—N1—C6	118.96 (18)	C2—C3—N3	118.8 (2)
N2—N1—H1	120.5	C4—C3—N3	119.1 (2)
C6—N1—H1	120.5	C2—C1—C6	120.0 (2)
C9—N2—N1	123.4 (2)	C2—C1—H1A	120.0
O4—C10—O5	122.7 (2)	C6—C1—H1A	120.0
O4—C10—C9	122.3 (2)	N2—C9—C10	122.4 (2)
O5—C10—C9	115.0 (2)	N2—C9—C8	113.5 (2)
C1—C6—C5	120.1 (2)	C10—C9—C8	124.1 (2)
C1—C6—N1	121.24 (19)	C8—C7—H7A	109.5
C5—C6—N1	118.64 (19)	C8—C7—H7B	109.5
O3—C8—C7	120.3 (2)	H7A—C7—H7B	109.5
O3—C8—C9	121.9 (2)	C8—C7—H7C	109.5
C7—C8—C9	117.8 (2)	H7A—C7—H7C	109.5
C4—C5—C6	119.8 (2)	H7B—C7—H7C	109.5
C4—C5—H5	120.1	O5—C11—H11A	109.5
C6—C5—H5	120.1	O5—C11—H11B	109.5
C3—C2—C1	119.2 (2)	H11A—C11—H11B	109.5
C3—C2—H2	120.4	O5—C11—H11C	109.5
C1—C2—H2	120.4	H11A—C11—H11C	109.5
C5—C4—C3	118.8 (2)	H11B—C11—H11C	109.5
C5—C4—H4	120.6		
			
C6—N1—N2—C9	180.0	O1—N3—C3—C4	180.0
N2—N1—C6—C1	0.0	C3—C2—C1—C6	0.0
N2—N1—C6—C5	180.0	C5—C6—C1—C2	0.0
C1—C6—C5—C4	0.0	N1—C6—C1—C2	180.0
N1—C6—C5—C4	180.0	N1—N2—C9—C10	0.0
C6—C5—C4—C3	0.0	N1—N2—C9—C8	180.0
O4—C10—O5—C11	0.0	O4—C10—C9—N2	0.0
C9—C10—O5—C11	180.0	O5—C10—C9—N2	180.0
C1—C2—C3—C4	0.0	O4—C10—C9—C8	180.0
C1—C2—C3—N3	180.0	O5—C10—C9—C8	0.0
C5—C4—C3—C2	0.0	O3—C8—C9—N2	180.0
C5—C4—C3—N3	180.0	C7—C8—C9—N2	0.0
O2—N3—C3—C2	180.0	O3—C8—C9—C10	0.0
O1—N3—C3—C2	0.0	C7—C8—C9—C10	180.0
O2—N3—C3—C4	0.0		

### Hydrogen-bond geometry (Å, °)

**Table:**

*D*—H···*A*	*D*—H	H···*A*	*D*···*A*	*D*—H···*A*
N1—H1···O4	0.86	1.98	2.618 (3)	130
C2—H2···O3^i^	0.93	2.55	3.279 (3)	135
C11—H11B···O1^ii^	0.96	2.57	3.128 (3)	117
N1—H1···O2^iii^	0.86	2.64	3.439 (3)	154
C5—H5···O2^iii^	0.93	2.62	3.467 (4)	153
C4^iii^—H4^iii^···O4	0.93	2.61	3.518 (3)	167

Symmetry codes: (i) −*x*+2, *y*−1/2, −*z*+1/2; (ii) *x*, *y*+1, *z*; (iii) −*x*+1, *y*+1/2, −*z*+1/2.
